# A virtual audit system for intensity‐modulated radiation therapy credentialing in Japan Clinical Oncology Group clinical trials: A pilot study

**DOI:** 10.1002/acm2.14040

**Published:** 2023-05-16

**Authors:** Mitsuhiro Nakamura, Dejun Zhou, Toshiyuki Minemura, Satoshi Kito, Hiroyuki Okamoto, Naoki Tohyama, Masahiko Kurooka, Yu Kumazaki, Masayori Ishikawa, Catharine H Clark, Elizabeth Miles, Joerg Lehmann, Nicolaus Andratschke, Stephen Kry, Satoshi Ishikura, Takashi Mizowaki, Teiji Nishio

**Affiliations:** ^1^ Department of Advanced Medical Physics Graduate School of Medicine Kyoto University Kyoto Japan; ^2^ Institute for Cancer Control National Cancer Center Tokyo Japan; ^3^ Department of Radiation Oncology Tokyo Metropolitan Cancer and Infectious Disease Center Komagome Hospital Tokyo Japan; ^4^ Radiation Safety and Quality Assurance Division National Cancer Center Hospital Tokyo Japan; ^5^ Division of Medical Physics Tokyo Bay Makuhari Clinic for Advanced Imaging Cancer Screening, and High‐Precision Radiotherapy Chiba Japan; ^6^ Department of Radiation Therapy Tokyo Medical University Hospital Tokyo Japan; ^7^ Department of Radiation Oncology International Medical Center Saitama Medical University Saitama Japan; ^8^ Faculty of Health Sciences Hokkaido University Hokkaido Japan; ^9^ National Radiotherapy Trials Quality Assurance (RTTQA) Group, Royal Surrey NHS Foundation Trust London UK; ^10^ Department of Radiotherapy Physics University College London Hospital London UK; ^11^ Department of Medical Physics and Bioengineering University College London London UK; ^12^ Medical Physics department National Physical Laboratory (NPL) Teddington UK; ^13^ National Radiotherapy Trials Quality Assurance (RTTQA) Group Mount Vernon Cancer Centre Northwood UK; ^14^ Trans Tasman Radiation Oncology Group (TROG) Newcastle Australia; ^15^ Department of Radiation Oncology Calvary Mater Hospital Newcastle Australia; ^16^ School of Information and Physical Sciences University of Newcastle Newcastle Australia; ^17^ Institute of Medical Physics University of Sydney Sydney Australia; ^18^ Department of Radiation Oncology University Hospital of Zurich University of Zurich Zurich Switzerland; ^19^ Imaging and Radiation Oncology Core (IROC) The University of Texas MD Anderson Cancer Center Houston Texas USA; ^20^ Division of Radiation Oncology Tokyo Bay Makuhari Clinic for Advanced Imaging Cancer Screening, and High‐Precision Radiotherapy Chiba Japan; ^21^ Department of Radiation Oncology and Image‐Applied Therapy Graduate School of Medicine Kyoto University Kyoto Japan; ^22^ Medical Physics Laboratory Division of Health Science Graduate School of Medicine Osaka University Osaka Japan

**Keywords:** gamma analysis, IMRT dosimetry credentialing, JCOG, prospective trials, virtual audit

## Abstract

**Purpose:**

The Medical Physics Working Group of the Radiation Therapy Study Group at the Japan Clinical Oncology Group is currently developing a virtual audit system for intensity‐modulated radiation therapy dosimetry credentialing. The target dosimeters include films and array detectors, such as ArcCHECK (Sun Nuclear Corporation, Melbourne, Florida, USA) and Delta4 (ScandiDos, Uppsala, Sweden). This pilot study investigated the feasibility of our virtual audit system using previously acquired data.

**Methods:**

We analyzed 46 films (32 and 14 in the axial and coronal planes, respectively) from 29 institutions. Global gamma analysis between measured and planned dose distributions used the following settings: 3%/3 mm criteria (the dose denominator was 2 Gy), 30% threshold dose, no scaling of the datasets, and 90% tolerance level. In addition, 21 datasets from nine institutions were obtained for array evaluation. Five institutions used ArcCHECK, while the others used Delta4. Global gamma analysis was performed with 3%/2 mm criteria (the dose denominator was the maximum calculated dose), 10% threshold dose, and 95% tolerance level. The film calibration and gamma analysis were conducted with in‐house software developed using Python (version 3.9.2).

**Results:**

The means ± standard deviations of the gamma passing rates were 99.4 ± 1.5% (range, 92.8%–100%) and 99.2 ± 1.0% (range, 97.0%–100%) in the film and array evaluations, respectively.

**Conclusion:**

This pilot study demonstrated the feasibility of virtual audits. The proposed virtual audit system will contribute to more efficient, cheaper, and more rapid trial credentialing than on‐site and postal audits; however, the limitations should be considered when operating our virtual audit system.

## 1 INTRODUCTION

In recent years, radiation therapy techniques have become more sophisticated, and prospective clinical trials are increasingly being conducted using highly advanced techniques, such as stereotactic body radiotherapy and intensity‐modulated radiation therapy (IMRT). As more institutions participate in clinical trials, ensuring consistent quality of treatment, especially for clinical trials involving radiation therapy, is important. According to a Trans‐Tasman Radiation Oncology Group report, a 20% difference in overall survival exists between compliant and noncompliant cohorts.^1^ Additionally, accurate and consistent dosimetry has been proven essential to ensure maximum new knowledge from clinical trials.^2^ These emphasize the importance of audits for institutions participating in clinical trials involving radiation therapy.

There are three types of audits for IMRT dosimetry credentialing: on‐site,^3^, ^4^ postal,^5^, ^6^ and virtual.^7^, ^8^, ^9^ During on‐site audits, external auditors visit each audited institution to perform dosimetry credentialing using their ionization chamber, electrometer, films, and phantoms. The auditors can confirm the measurement process, including the institutional dose calibration protocol and immediate results, and hold discussions; however, the disadvantages include associated travel costs and long working hours. Postal audits are more efficient than on‐site audits.^5^, ^6^ A phantom containing small dosimeters, such as radiophotoluminescent glass dosimeters, thermoluminescent dosimeters, and films, is mailed to the participating institutions. After performing the entire procedure, the local staff returns the phantom to the auditors who analyze the results; this is the main methodology for IMRT dosimetry credentialing for Japan Clinical Oncology Group (JCOG) and the Imaging and Radiation Oncology Core (IROC) trials. However, the number of phantom sets is limited, and resolving discrepancies is challenging because the auditors are not involved in the irradiation process. Recently, the European Organization for the Research and Treatment of Cancer (EORTC) and an Australian group established their virtual audit systems for films, array detectors, and electronic portal imaging devices.^7^, ^8^, ^9^ Unlike a postal audit, a virtual audit does not require the auditors to mail the phantom set because the equipment used for measurement belongs to the audited institution. Moreover, the analysis is performed by the auditors and not the local staff. Thus, independence is achieved for the auditing site performing the analysis. A virtual audit is expected to be more efficient, cheaper, and have more rapid case enrollment than on‐site and postal audits.

The Medical Physics Working Group of the Radiation Therapy Study Group at the JCOG is developing a virtual audit system to replace the current postal audit system to satisfy the increasing IMRT dosimetry credentialing requirements. We assume that the target dosimeters of the virtual audit are films and array detectors, such as ArcCHECK (Sun Nuclear Corporation, Melbourne, Florida, USA) and Delta4 (ScandiDos, Uppsala, Sweden). This study investigated the feasibility of our virtual audit system using previously acquired data.

## 2 MATERIALS AND METHODS

### 2.1 Proposed workflow of our virtual audit

**FIGURE 1:**
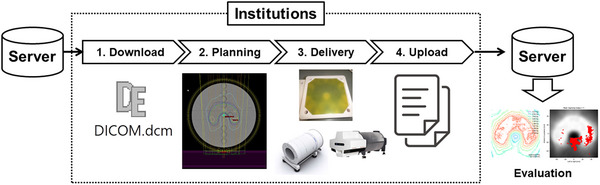
Workflow of our virtual audit.

**FIGURE 2:**
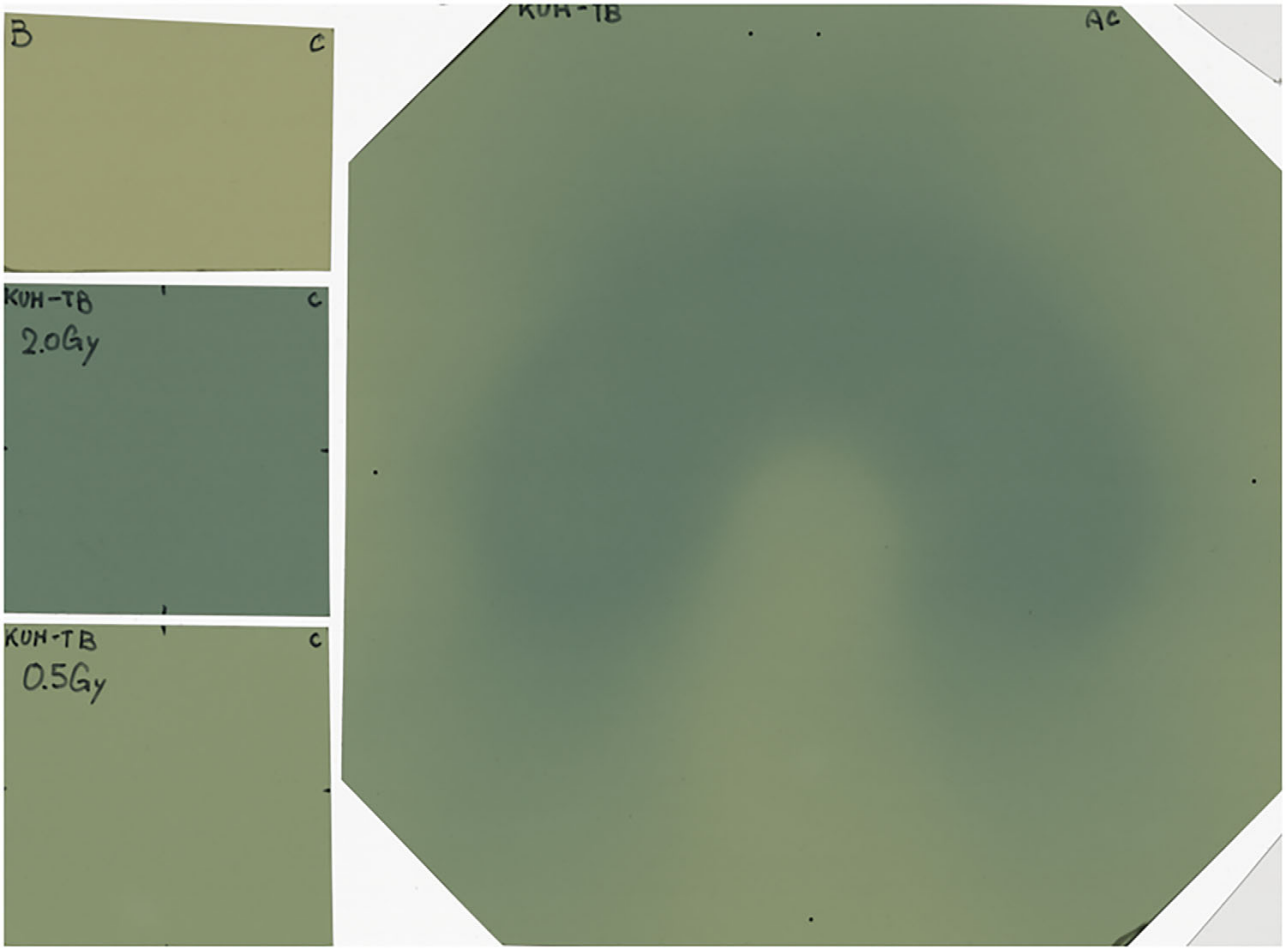
Example of scan file including an irradiated film and three subfilms.

The proposed workflow of our virtual audit for IMRT dosimetry credentialing is illustrated in Figure 1. First, the audited institutions download computed tomography (CT) images and structure datasets of the audit phantom in a DICOM‐RT format from a dedicated server and import them into their treatment planning system. Second, the institution designs an IMRT treatment plan satisfying the following dose‐volume constraints^4^: (1) a prescription dose of 20 Gy in 10 fractions should cover 95% of the C‐shaped planning target volume (PTV). (2) The maximum doses to the PTV and surrounding organ‐at‐risk should be less than 110% and 60%, respectively. (3) The local maximum dose should be within the PTV. Third, the dose distribution is recalculated on their patient‐specific quality assurance (QA) phantom without changing the treatment parameters (referred to as the planned dose distribution). Simultaneously, the audited institutions transfer the treatment plan information to their treatment machine and deliver a single fraction to their phantom as planned (referred to as the measured dose distribution). Fourth, the audited institution uploads the measured and planned dose distributions in the specified file and DICOM‐RT formats, respectively, to our server without modifying the obtained measurement data. For institutions delivering a fractional dose to films, at least three subfilms, 0, 0.5, and 2 Gy (or more if necessary), are necessary to obtain a calibration curve using the third‐degree polynomial regression.^10^ According to the instructions, the institutions are requested to upload one scan file, including an irradiated film and three subfilms, as the measured dose distribution. Figure 2 shows an example of the film layout. However, for institutions delivering a fractional dose to the array detectors, the measured dose distribution is uploaded in a text format. Finally, the film calibration (when necessary) and gamma analysis are automatically performed by the auditors using in‐house software developed using Python (version 3.9.2) and hosted on the server.

### 2.2 Film evaluation

This study analyzed previously scanned data from postal audits. The planned dose was delivered to the water‐equivalent phantom containing a film in axial or coronal planes. We used 46 films (32 and 14 in the axial and coronal planes, respectively) from 29 institutions. Among them, four institutions tested two treatment machines each. We obtained eight film data from the four institutions. Table 1 summarizes the combination of the treatment machine and planning system.

**TABLE 1:** Summary of the combinations of treatment machine and treatment planning system in film analysis.

Machine	TPS	Number (%)
Halcyon	Eclipse	3 (9.4)
iX	Eclipse	4 (12.5)
Novalis	Eclipse	2 (6.3)
ONCOR	Eclipse	1 (3.1)
TrueBeam	Eclipse	8 (25.0)
TrueBeam Edge	Eclipse	2 (6.3)
TrueBeam STx	Eclipse	2 (6.3)
iX	Pinnacle	1 (3.1)
Synergy	Pinnacle	1 (3.1)
Infinity	Monaco	1 (3.1)
Synergy	Monaco	1 (3.1)
Versa HD	Monaco	2 (6.3)
Radixact	Presicion	1 (3.1)
Tomotherapy	Planning Station	1 (3.1)
TrueBeam	RayStation	1 (3.1)
Versa HD	RayStation	1 (3.1)

Abbreviation: TPS, treatment planning system.

The details of the scanned data are as follows. Three subfilms for the calibration curve were acquired by placing a film at a depth of 10 cm for a source‐to‐axis distance of 100 cm. The films were irradiated perpendicular to the beam axis with a field size of 100 × 100 mm^2^ at a gantry angle of 0°. The reference setup for the calibration film is shown in Figure S1. A dataset, including the irradiated and three subfilms, was scanned (Figure 2). According to AAPM TG235, the scanned optical densities are not uniform across the scanner^11^; therefore, they were corrected by subtracting the optical density obtained with the unset film.

The developed in‐house software read the scan data with a resolution of 150 dpi in a 48‐bit RGB format and extracted the red channel data without applying smoothing or filtering.^5^ The calibration curve was then automatically generated from the three subfilms using a third‐degree polynomial regression.^10^ The isocenter was automatically identified from the four points marked near each side, and the rotation was corrected. Global gamma analysis was conducted between the measured and planned dose distributions,^12^ using coding as in.^13^ The following settings were used for film evaluation: 3%/3 mm criteria (the dose denominator was 2 Gy), 30% threshold dose, no dataset scaling, and 90% tolerance level. These settings have been determined after careful consideration.^3^

The gamma passing rates calculated with Simple QA Analysis (Triangle Products, Chiba, Japan) used in the postal audit were compared with those from our procedure. In the postal audit, a calibration curve was obtained by modifying the reference calibration curve based on the irradiated dose. In contrast, this study does not compare the reference calibration curve with that generated using the in‐house software. The other settings were identical for both procedures.

### 2.3 Array detector evaluation

Array detectors are assumed as one of the target dosimeters. Therefore, we acquired 21 datasets from nine institutions for array detector evaluation. Table 2 summarizes the combination of the treatment machine, treatment planning system, and array detectors. Five institutions used ArcCHECK, whereas the others used Delta4. The detectors were arranged in a helical array with a spacing of 1 cm for ArcCHECK. In contrast, the detectors in Delta4 were built in two orthogonal planes with a resolution of 0.5 cm in the central 6 × 6 cm^2^ region and 1 cm in the outer region.

**TABLE 2:** Summary of the combinations of treatment machine, treatment planning system, and array detectors.

Machine	TPS	Array detector	Number (%)
TrueBeam STx	Eclipse	ArcCHECK	2 (9.5)
VitalBeam	Eclipse	ArcCHECK	1 (4.8)
TrueBeam	Eclipse	ArcCHECK	3 (14.3)
TrueBeam	RayStation	ArcCHECK	2 (9.5)
iX	Eclipse	ArcCHECK	1 (4.8)
Versa HD	RayStation	ArcCHECK	1 (4.8)
Versa HD	Monaco	ArcCHECK	1 (4.8)
TrueBeam	Eclipse	Delta4	4 (19.0)
TrueBeam STx	Eclipse	Delta4	4 (19.0)
Radixact	Presicion	Delta4	1 (4.8)
Halcyon	Eclipse	Delta4	1 (4.8)

Abbreviation: TPS, treatment planning system.

The in‐house software reads the DICOM‐RT PLAN, DOSE files, and measured data in a text format. For ArcCHECK, planned dose distributions were acquired from Refs.^14^, ^15^ In contrast, planned dose distributions in the coronal and sagittal planes at the isocenter level were extracted for Delta4. Global gamma analysis was performed with 3%/2 mm criteria (the dose denominator was the maximum calculated dose), 10% threshold dose, and 95% tolerance level based on the AAPM recommendation.^16^ The gamma passing rates for the reconstructed detector planes for ArcCHECK and Delta4 were calculated using the in‐house software by referring to Ref.^13^ For comparison, gamma passing rates were precalculated using local dedicated commercial software at the audited institutions.

### 2.4 Statistical analysis

The gamma passing rates from the postal audit were statistically compared with those from our film evaluation procedure. In addition, the gamma passing rates calculated using the local dedicated commercial and in‐house software were compared for array detector evaluation. The paired *t*‐test with a 5% significance level was used for statistical analyses.

## 3 RESULTS

### 3.1 Film evaluation

**FIGURE 3:**
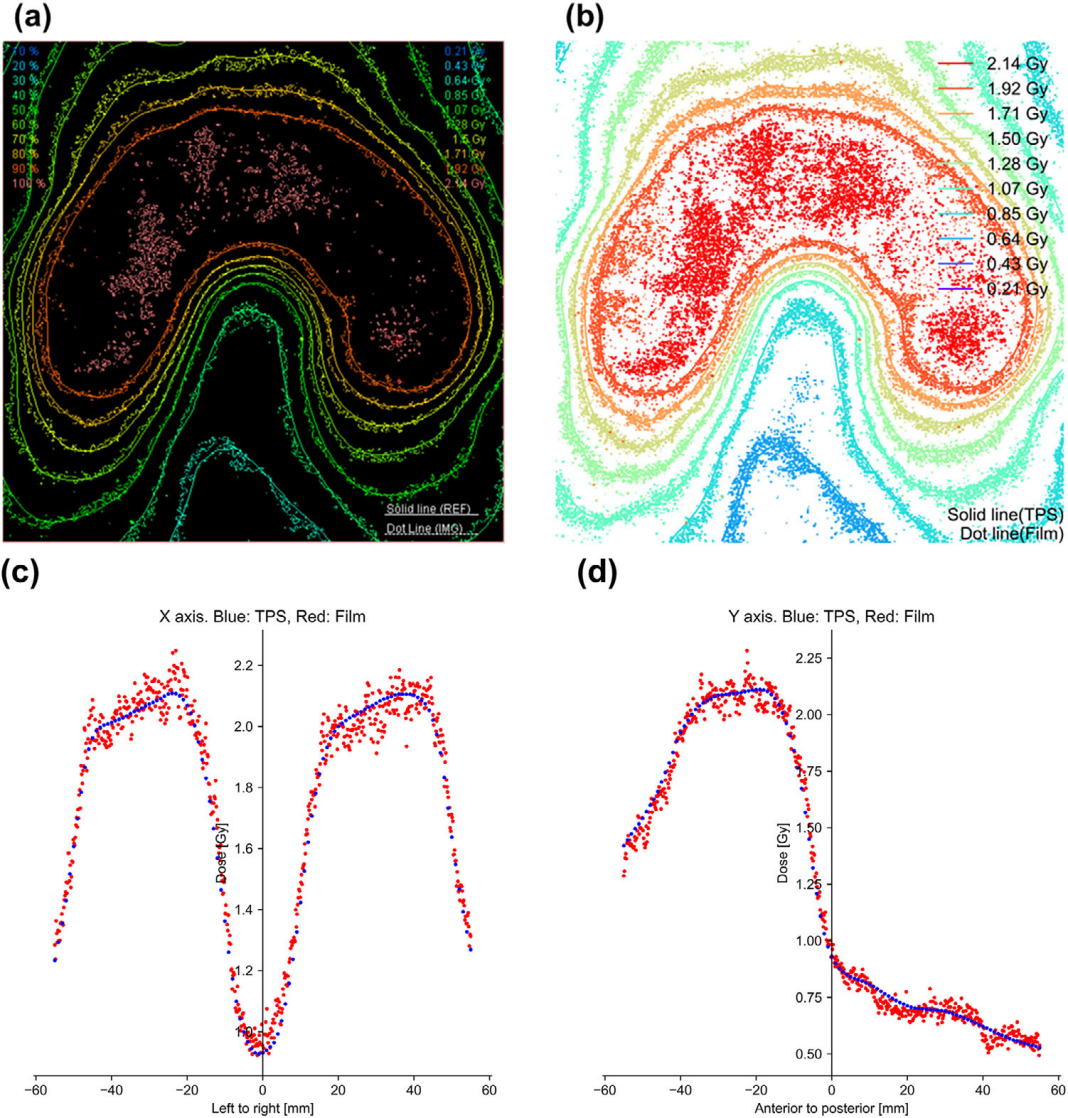
Example of the results of film dosimetry. (a) Postal audit procedure, (b) proposed procedure, (c) dose profile in lateral direction, and (d) dose profile in vertical direction.

The means ± standard deviations (SD) of the gamma passing rates were 97.7 ± 2.5% (range, 92.3%–100%) for the postal audit and 98.6 ± 4.2% (range, 74.0%–100%) for our procedure. Figure 3 shows an example of the film dosimetry results. Two films, with gamma passing rates of 89.9 and 74.0% in our procedure, failed to meet the tolerance level of 90% (the reasons are mentioned in the Discussion section). After omitting these two films, our procedure's mean ± SD of the gamma passing rates was 99.4 ± 1.5% (range, 92.8%–100%). There was a statistically significant difference in the gamma passing rates between the postal audit and our procedure (*p* < 0.05).

### 3.2 Array detector evaluation

Table 3 summarizes the mean ± SD of the gamma passing rates using the local dedicated commercial and in‐house software. The gamma passing rates were more than 95%, and no significant difference was observed between the two results (*p* = 0.813).

**TABLE 3:** Summary of the gamma passing rates with the local dedicated commercial software and in‐house‐developed software in array evaluation.

	Local dedicated commercial software	In‐house developed software	*p*‐value
ArcCHECK (*N* = 11)	98.7 ± 0.9% (range: 97.4%–100%)	98.9 ± 0.9% (range: 97.6%–100%)	0.643
Delta4 (*N* = 10)	99.7 ± 0.8% (range: 97.3%–100%)	99.6 ± 1.0% (range: 97.0%–100%)	0.619
All (*N* = 21)	99.2 ± 1.0% (range: 97.3%–100%)	99.2 ± 1.0% (range: 97.0%–100%)	0.813

## 4 DISCUSSION

### 4.1 Film evaluation

Two films showed gamma passing rates lower than 90% with our procedure. Interviews conducted at the auditing site revealed that two institutions had mistakenly delivered subfilms with a dose greater than 2 Gy (2.17 and 2.293 Gy, respectively). In the postal audit, all film dosimetry results met the tolerance level of 90% because the calibration curve was generated according to the irradiated dose. In contrast, our procedure could successfully detect such deficiencies during film evaluation. After omitting these two films, there was a statistically significant difference in the gamma passing rates between the postal audit and our procedure (*p* < 0.05), attributed to the calibration curve. As described in the Materials and methods section, in a postal audit, a reference calibration curve, prepared in advance, is tuned based on the doses of 0.5 and 2 Gy on the subfilms. However, our calibration curve is directly generated by the subfilms using the third‐degree polynomial regression without a reference calibration curve. Furthermore, the statistical difference may have occurred because of the implementation of the gamma calculation. Hussein et al. revealed that different devices and software combinations exhibit varying levels of agreement even for the same underlying data.^17^, ^18^ Despite these differences, the results of the virtual audit procedure were consistent with those of the previous postal audit.^3^

### 4.2 Array detector evaluation

Diode spacing is the main concern in dose distribution analysis using array detectors. The array detectors have a spatial resolution lower than that of the films.^7^, ^19^ Weber et al. showed a large inconsistency in the gamma analysis results between the postal audit by IROC and virtual audit by EORTC, especially at institutions using Delta4.^7^ Although they pointed to a low resolution as the cause of the discrepancy, no such trend was observed in this study. For ArcCHECK, the active area of each diode was 0.64 mm^2^, which is sufficient for a treatment planning system with the highest resolution of 1 mm. Possible causes for the discrepancy may include the inappropriate assignment of HU values or media to the phantom for array detectors and not the resolution.

The gamma analysis criteria in array detector evaluation were different from those in film evaluation. We found that the gamma passing rate was exceptionally high (always almost 100%) in the array detector evaluation, when applying the same criteria as the film evaluation criteria. Therefore, we apply the AAPM recommendation, which is more stringent than the film evaluation criteria, in array detector evaluation.

### 4.3 IMRT treatment plan for dosimetry audit

A common IMRT treatment plan is designed to satisfy the dose‐volume constraints for IMRT dosimetry audit. Prior to patient enrollment, participating institutions are also subject to a dummy run study involving protocol‐specific dose‐volume constraints for any specific clinical trial to assess compliance with treatment planning.^20^ Moreover, a specific clinical trial requires additional tests, such as small field dosimetry and audit for the image‐guided radiotherapy.^21^, ^22^ Thus, we determined the suitability of the institution by considering all the above factors.

### 4.4 Operation design of virtual audit for IMRT dosimetry credentialing

The proposed virtual audit operational design is as follows: institutions first submit the data from the array detectors. If the result does not pass the IMRT dosimetry credentialing following careful reviewing by the auditing group, the institutions are asked to submit the film dosimetry dataset. If institutions fail the film‐based virtual audit because of technical problems, we conduct a postal audit. As all audited institutions passed the postal audit based on our auditing experience, the proposed operational design is feasible.

Film and array detector results are relative dose distribution checks and do not directly provide adequate information on the absolute dose calibrations. Therefore, beam output audits are additionally required.^23^ Clinically unacceptable errors in the reference output doses would impact the clinical outcomes. In Japan, most institutions, including those participating in the JCOG trial, request the Association for Nuclear Technology in Medicine (ANTM) to measure the reference output dose.^24^ We require participating institutions to measure the reference output dose by ANTM and submit the results in the virtual audit.

### 4.5 Reason for not including planar array detectors

Our virtual audit system does not support an analysis using planar array detectors at present. The Japan Society of Medical Physics QA/QC committee conducted a web‐based survey on patient‐specific QA for IMRT and obtained responses from 148 institutions in Japan.^25^ Array detectors were the most popular devices used for dose distribution measurements at 107 institutions (72.3%), followed by films at 23 institutions (15.5%) and planar array detectors (10.1%). Over 85% of the institutions performing IMRT use non‐planar array detectors or films. In addition, even if institutions do not have array detectors, they can perform film dosimetry, as described by the IMRT guidelines in Japan,^26^ and can be virtually audited using films. As many planar array detectors exist, evaluating and managing them is difficult because of the various file formats. In contrast, film dosimetry is time‐consuming and prone to user errors. In the future, we will establish the virtual audit system for planar array detectors, and then request each institution to submit their results from a planar array detector of their choice, with film results as a secondary check.

### 4.6 Substitutability of the audit process with institutional patient‐specific QA

Participating institutions are expected to halt case enrollment until the audit results are available. We discussed the feasibility of replacing the audit with institutional patient‐specific QA results to shorten the waiting period. However, Kry et al. quantitatively demonstrated that institutional patient‐specific QA is not a reasonable substitute for audit.^27^ The omission of an audit may miss any under‐ or over‐dosage, thus compromising the quality of prospective clinical trial outcomes. Therefore, maintaining the audit system in prospective clinical trials is essential.

### 4.7 Limitations

First, we scanned the film using 150 dpi, based on our previous study.^5^ Huang et al. investigated the sensitivity of the gamma index to the film resolution in the measured dose distribution using three different resolutions (71, 142, and 285 dpi).^28^ They stated that a high resolution can artificially increase the percentage of pixels with passing gamma values. As shown in Table S1, however, we confirmed film resolutions negligibly effect gamma analysis results. Second, this study showed that passing results are just passing, especially for array detector evaluation. From a sensitivity and specificity perspective, further research is necessary to validate this approach, considering the failing results. Third, the virtual audit uses partially independent equipment and is more likely to overlook some problems. Hence, in addition to submitting the audit results, further investigation, such as the assignment of HU values or media, is necessary for actual operation because institutions may miscalibrate their array detectors to absorb systematic errors. Fourth, the institution can first compare the results before submitting them. However, as the IMRT dosimetry audit criteria are unavailable to the local staff, they cannot know the criteria for evaluation.

## 5 CONCLUSION

We investigated the feasibility of our virtual audit system using previously acquired data. This pilot study demonstrated the feasibility of virtual audits. The virtual audit system will provide more efficient, cheaper, and more rapid trial credentialing than on‐site and postal audits. However, the limitations should be considered when operating our virtual audit system.

## AUTHOR CONTRIBUTIONS

Mitsuhiro Nakamura, Toshiyuki Minemura, Satoshi Kito, Hiroyuki Okamoto, Naoki Tohyama, Masahiko Kurooka, Yu Kumazaki, Masayori Ishikawa and Teiji Nishio planned the study. Mitsuhiro Nakamura and Dejun Zhou performed the statistical analysis and drafted the manuscript. Mitsuhiro Nakamura, Toshiyuki Minemura, Satoshi Kito, Hiroyuki Okamoto, Naoki Tohyama, Masahiko Kurooka, Yu Kumazaki, Masayori Ishikawa and Teiji Nishio conceived the study and participated in its design and coordination. Catharine H Clark, Elizabeth Miles, Joerg Lehmann, Nicolaus Andratschke, and Stephen Kry helped draft the manuscript. All authors read and approved the final manuscript.

## CONFLICT OF INTEREST STATEMENT

We have no financial relationships to disclose.

## Supporting information

Supporting Information

Supporting Information

## Data Availability

Data available on request from the authors.

## References

[1] Peter L , O'Sullivan B , Giralt J , et al. Critical impact of radiotherapy protocol compliance and quality in the treatment of advanced head and neck cancer: results from TROG 02.02. J Clin Oncol. 2010;28:2996‐3001. 20479390 10.1200/JCO.2009.27.4498

[2] Pettersen M , Aird E , Olsen D . Quality assurance of dosimetry and the impact on sample size in randomized clinical trials. Radiother Oncol. 2008;86:195‐199. 17727987 10.1016/j.radonc.2007.07.001

[3] Nishio T , Nakamura M , Okamoto H , et al. An overview of the medical‐physics‐related verification system for radiotherapy multicenter clinical trials by the Medical Physics Working Group in the Japan Clinical Oncology Group‐Radiation Therapy Study Group. J Radiat Res. 2020;61:999‐1008. 32989445 10.1093/jrr/rraa089 PMC7674673

[4] Nakamura M , Minemura T , Ishikura S , et al. An on‐site audit system for dosimetry credentialing of intensity‐modulated radiotherapy in Japanese clinical oncology group (JCOG) clinical trials. Phys Med. 2016;32:987‐991. 27402255 10.1016/j.ejmp.2016.07.002

[5] Okamoto H , Minemura T , Nakamura M , et al. Establishment of postal audit system in intensity‐modulated radiotherapy by radiophotoluminescent glass dosimeters and a radiochromic film. Phys Med. 2018;48:119‐126. 29728224 10.1016/j.ejmp.2018.03.013

[6] Carson M , Molineu A , Taylor P , et al. Examining credentialing criteria and poor performance indicators for IROC Houston's anthropomorphic head and neck phantom. Med Phys. 2016;43:6491‐6496. 27908168 10.1118/1.4967344 PMC5106427

[7] Weber D , Vallet V , Molineu A , et al. IMRT credentialing for prospective trials using institutional virtual phantoms: results of a joint European Organization for the research and treatment of cancer and radiological physics center project. Radiat Oncol. 2014;9:123. 24885438 10.1186/1748-717X-9-123 PMC4046849

[8] Miri N , Lehmann J , Legge K , et al. Virtual EPID standard phantom audit (VESPA) for remote IMRT and VMAT credentialing. Phys Med Biol. 2017;62:4293‐4299. 28248642 10.1088/1361-6560/aa63df

[9] Miri N , Lehmann J , Legge K , et al. Remote dosimetric auditing for intensity modulated radiotherapy: a pilot study. Phys Imaging Radiat Oncol. 2017;4:26‐31.

[10] Robatjazi M , Mahdavi S , Takavr A , et al. Application of Gafchromic EBT2 film for intraoperative radiation therapy quality assurance. Phys Med. 2015;31:314‐319. 25703011 10.1016/j.ejmp.2015.01.020

[11] Niroomand‐Rad A , Sou‐Tung C , Gram M , et al. Report of AAPM task group 235 radiochromic film dosimetry: an update to TG‐55. Med Phys. 2020;47:5986‐6025. 32990328 10.1002/mp.14497

[12] Low D , Harms W , Mutic S , et al. A technique for the quantitative evaluation of dose distributions. Med Phys. 1998;25:656‐661. 9608475 10.1118/1.598248

[13] Pipek J , Calculation of gamma index on two matrices of the same shape. https://gist.github.com/janpipek/334c2533b87cd75c3f59

[14] Peet S , Flash Gamma. Accessed November 1, 2022. https://github.com/samuelpeet/flashgamma

[15] Peet S , Yu L , Maxwell S , et al. Exploring the gamma surface: a new method for visualising modulated radiotherapy quality assurance results. Phys Med. 2020;78:166‐172. 33035928 10.1016/j.ejmp.2020.09.021

[16] Miften M , Olch A , Mihailidis D , et al. Tolerance limits and methodologies for IMRT measurement‐based verification QA: recommendations of AAPM task group no. 218. Med Phys. 2018;45:e53‐83. 29443390 10.1002/mp.12810

[17] Hussein M , Rowshanfarzad P , Ebert M , et al. A comparison of the gamma index analysis in various commercial IMRT/VMAT QA systems. Radiother Oncol. 2013;109:370‐376. 24100148 10.1016/j.radonc.2013.08.048

[18] Hussein M , Clementel E , Eaton D , et al. A virtual dosimetry audit – towards transferability of gamma index analysis between clinical trial QA groups. Radiother Oncol. 2017;125:398‐404. 29100698 10.1016/j.radonc.2017.10.012

[19] Fredh A , Scherman J , Fog L , et al. Patient QA systems for rotational radiation therapy: a comprehensive experimental study with intentional errors. Med Phys. 2013;40:031716. 23464311 10.1118/1.4788645

[20] Okamoto H , Murakami N , Isohashi F , et al. Dummy‐run for standardizing plan quality of intensity‐modulated radiotherapy for postoperative uterine cervical cancer: Japan Clinical Oncology Group study (JCOG1402). Radiat Oncol. 2019;14:133. 31358026 10.1186/s13014-019-1340-y PMC6664568

[21] Kumazaki Y , Ozawa S , Nakamura M , et al. An end‐to‐end postal audit test to examine the coincidence between the imaging isocenter and treatment beam isocenter of the IGRT linac system for Japan Clinical Oncology Group (JCOG) clinical trials. Phys Med. 2018;53:145‐152. 30241749 10.1016/j.ejmp.2018.08.010

[22] Kimura T , Nagata Y , Eba J , et al. A randomized Phase III trial of comparing two dose‐fractionations stereotactic body radiotherapy (SBRT) for medically inoperable Stage IA non‐small cell lung cancer or small lung lesions clinically diagnosed as primary lung cancer: japan Clinical Oncology Group Study JCOG1408 (J‐SBRT trial). Jpn J Clin Oncol. 2017;47:277‐281. 28073946 10.1093/jjco/hyw198

[23] Kry S , Peterson C , Howell R , et al. Remote beam output audits: a global assessment of results out of tolerance. Phys Imaging Radiat Oncol. 2018;7:39‐44. 31872085 10.1016/j.phro.2018.08.005 PMC6927685

[24] Mizuno H , Yamashita W , Okuyama H , et al. Analysis of the uncertainties in the dose audit system using radiophotoluminescent glass dosimeters in Japanese radiotherapy units. Radiat Meas. 2022;153:106753.

[25] Anetai Y , Sumida I , Kumazaki Y , et al. Assessment of using a gamma index analysis for patient‐specific quality assurance in Japan. J Appl Clin Med Phys. 2022;23:e13745. 36018627 10.1002/acm2.13745 PMC9588274

[26] https://www.jastro.or.jp/customer/guideline/2016/10/IMRT2011.pdf

[27] Kry S , Molineu A , Kerns J , et al. Institutional patient‐specific IMRT QA does not predict unacceptable plan delivery. Int J Radiat Oncol Biol Phys. 2014;90:1195‐1201. 25442044 10.1016/j.ijrobp.2014.08.334 PMC4276500

[28] Huang J , Pulliam K , McKenzie E , et al. Effects of spatial resolution and noise on gamma analysis for IMRT QA. J Appl Clin Med Phys. 2014;15:93‐104. 25207399 10.1120/jacmp.v15i4.4690 PMC4283459

