# Supplementary figures and images for: A virtual audit system for intensity‐modulated radiation therapy credentialing in Japan Clinical Oncology Group clinical trials: A pilot study
Source: J Appl Clin Med Phys. 2023 May 16;24(6):e14040. doi: 10.1002/acm2.14040 (PMC10243313; doi:10.1002/acm2.14040)

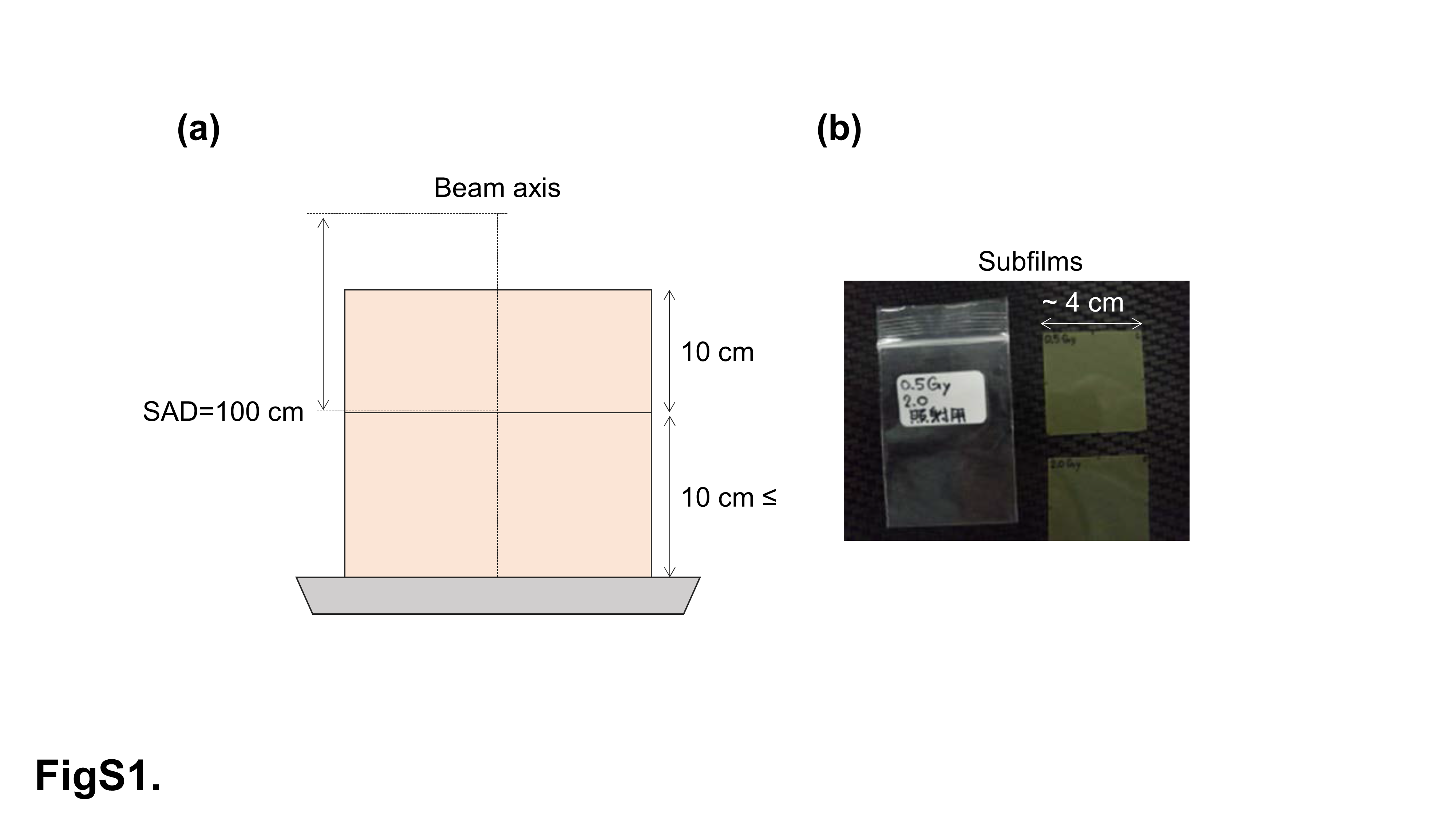

Supplement: Supplementary file 1 — Supporting Information [file ACM2-24-e14040-s002.tif]
