# Supplementary material for: A virtual audit system for intensity‐modulated radiation therapy credentialing in Japan Clinical Oncology Group clinical trials: A pilot study
Source: J Appl Clin Med Phys. 2023 May 16;24(6):e14040. doi: 10.1002/acm2.14040 (PMC10243313; doi:10.1002/acm2.14040)
Supplement: Supplementary file 2 — Supporting Information [file ACM2-24-e14040-s001.docx]

**Supplementary Table 1.** Comparison of the gamma passing rates between 150 dpi and 75 dpi with in-house-developed software. The following settings were used: 3%/3 mm criteria (the dose denominator was 2 Gy), 30% threshold dose, and no dataset scaling.

| **Film ID** | **150 dpi [%]** | **75 dpi [%]** | **Difference [%pts.]** |
| --- | --- | --- | --- |
| 1 | 100.0 | 99.7 | 0.3 |
| 2 | 100.0 | 100.0 | 0.0 |
| 3 | 100.0 | 100.0 | 0.0 |
| 4 | 100.0 | 100.0 | 0.0 |
| 5 | 99.8 | 99.5 | 0.3 |
| 6 | 100.0 | 100.0 | 0.0 |
| 7 | 98.8 | 98.3 | 0.5 |
| 8 | 100.0 | 100.0 | 0.0 |
| 9 | 100.0 | 100.0 | 0.0 |
| 10 | 100.0 | 100.0 | 0.0 |
| 11 | 99.9 | 99.9 | 0.0 |
| 12 | 99.1 | 99.3 | -0.2 |
| 13 | 100.0 | 100.0 | 0.0 |
| 14 | 100.0 | 100.0 | 0.0 |
| 15 | 100.0 | 100.0 | 0.0 |
| 16 | 97.6 | 96.2 | 1.4 |
| 17 | 100.0 | 99.8 | 0.2 |
| 18 | 100.0 | 100.0 | 0.0 |
| 19 | 99.0 | 98.8 | 0.2 |
| 20 | 100.0 | 100.0 | 0.0 |
| 21 | 100.0 | 100.0 | 0.0 |
| 22 | 100.0 | 99.1 | 0.9 |
| 23 | 100.0 | 100.0 | 0.0 |
| 24 | 100.0 | 100.0 | 0.0 |
| 25 | 99.0 | 100.0 | -1.0 |
| 26 | 98.9 | 97.4 | 1.5 |
| 27 | 100.0 | 100.0 | 0.0 |
| 28 | 99.4 | 98.8 | 0.6 |
| 29 | 96.6 | 92.5 | 4.1 |
| 30 | 100.0 | 100.0 | 0.0 |
| 31 | 100.0 | 100.0 | 0.0 |
| 32 | 100.0 | 100.0 | 0.0 |
| 33 | 99.5 | 98.2 | 1.3 |
| 34 | 10.0 | 100.0 | 0.0 |
| 35 | 100.0 | 99.9 | 0.1 |
| 36 | 99.9 | 99.8 | 0.1 |
| 37 | 100.0 | 100.0 | 0.0 |
| 38 | 99.8 | 99.8 | 0.0 |
| 39 | 100.0 | 100.0 | 0.0 |
| 40 | 100.0 | 100.0 | 0.0 |
| 41 | 99.9 | 99.9 | 0.0 |
| 42 | 92.8 | 87.8 | 5.0 |
| 43 | 99.9 | 99.7 | 0.2 |
| 44 | 93.6 | 89.6 | 4.0 |
| 45 | 89.8 | 90.2 | -0.4 |
| 46 | 74.0 | 72.5 | 1.5 |
| Mean | 98.6 | 98.2 | 0.4 |
| SD | 4.2 | 4.8 | 1.2 |
